# Experimental infection of acute bee paralysis virus induces differential responses of selected honey bee immune genes, independent of varroa resistance

**DOI:** 10.1038/s41598-026-65201-4

**Published:** 2026-08-05

**Authors:** Xinyan Ruan, Amélie Noël, Srinivas Thaduri, Joachim R. de Miranda, Barbara Locke

**Affiliations:** 1 Honey Bee Research Centre, Department of Ecology, Swedish University of Agricultural Sciences, Uppsala, 756 51 Sweden; 2 Surabhi Institute of Medical Science, Siddipet, 502375 Telananga India

**Keywords:** *Apis mellifera*, Insect immunity, *acute bee paralysis virus*, *Varroa destructor*, Varroa resistance, Virus tolerance, Immunology, Microbiology

## Abstract

Honeybees (*Apis mellifera*) have a complex immune system, with both innate and social immunity. The mite-resistant honeybee population from Gotland, Sweden, is well-known for their independent resistance to *Varroa destructor* mites and tolerance to virus infections, particularly *deformed wing virus* and *acute bee paralysis virus* (ABPV). However, the immune mechanisms underlying their virus tolerance remains unclear. Adult bees from this mite-resistant (MR) and a mite-susceptible (MS) control population were orally inoculated with either ABPV or a mock inoculum, in a laboratory time-course experiment. Two representative genes from five major immune signalling pathways, previously shown to respond to viral infections, were quantified using RT-qPCR. The genes exhibited distinct responses to ABPV inoculation, with some genes showing clear treatment-related differential expression between 24 and 48 h post-inoculation while other genes remained unaffected. However, there was no difference in the expression patterns between MR and MS populations. These findings suggest that the enhanced virus tolerance of the Gotland mite-resistant population is not due to differential regulation of the immune genes examined here. They also highlight the dynamic nature of immune gene expression, refine hypotheses for further studies of honeybee virus tolerance and motivate more comprehensive molecular analyses of a selection of these samples.

**Supplementary Information:**

The online version contains supplementary material available at 10.1038/s41598-026-65201-4.

## Introduction

Honeybees are one of the most important pollinators of cultivated and wild plants world-wide, providing a vital ecosystem service essential to both natural plant biodiversity and food production systems. However, several categories of diseases exert significant pressure on honeybee health, leading to population declines threatening their economic value through impacts on beekeeping and pollination service. One particularly destructive parasite, *Varroa destructor*, has spread globally causing honeybee colony collapse at unprecedented rates^1^. The immediate cause of varroa-mediated mortality is infection by viruses that are efficiently transmitted by varroa mites. For example, *deformed wing virus* (DWV) and *acute bee paralysis virus* (ABPV) are among the most extensively studied and epidemiologically important varroa-associated viruses having been implicated in colony losses worldwide^2–4^. To defend themselves against these infections, honeybees rely on two types of immune defense: social immunity, which involves community-level mechanisms such as the selective removal of infected individuals^5,6^, and individual innate immunity, which includes molecular^7–9^ and physiological defense responses^10–12^.

Innate immunity, consisting of humoral and cellular responses, is ancient and conserved across all organisms. In the humoral response, pathogen recognition triggers the activation of several signalling pathways, including Toll, Imd, JNK and JAK/STAT. The Toll and Imd pathways are two NF-κB signalling cascades that coordinate the production of antimicrobial peptides (AMPs). More than 150 types of AMPs have been identified in insects^13^, the vast majority of whom rely heavily on individual humoral immunity to protect themselves against environmental stressors. However, honeybees have only one-third of the immune genes found in *Drosophila*, including a vastly reduced suite of AMPs compared to the seven families of AMPs found in fruit flies^14,15^. In honeybees, the humoral response mainly induces the synthesis of four kinds of AMPs: hymenoptaecins, apidaecins, defensins and abaecins^16–18^. In addition to the Toll and Imd pathways, which are primarily associated with defense against bacterial and fungal infections, the JAK/STAT pathway plays a broader role in responding to various types of pathogens^19^. Although its effect on innate immunity is indirect, the influence of the JAK/STAT pathway on innate immunity is essential, contributing to processes such as cell growth, differentiation, homeostasis, and apoptosis^19^. The RNA interference (RNAi) pathway is a universal molecular mechanism for regulating the targeted turnover of individual mRNA species, and as such the most proximate and direct molecular defense against RNA viruses, which are susceptible to RNAi-mediated destruction during replication^20^. Key components of the RNAi pathway include Dicer, Argonaute, SID and Pasha^21–23^. RNAi based pesticides can provide molecular precision to pest management in crop production, vector control, and pollinator protection^24,25^ and has been shown to be effective against both honeybee viruses^26,27^ and varroa^28,29^.

Honeybees are highly susceptible to varroa-transmitted virus infections and beekeepers use a variety of chemical treatments and management strategies to reduce the mite population growth in the colonies and avoid the establishment of a virus epidemic, which would typically kill the colony within a couple of years^1^. However, several honeybee populations worldwide have demonstrated long-term survival, despite uncontrolled mite infestation^30^ and more such populations continue to be identified, both managed and free-living^31–34^. One such population, on an isolated peninsula of the Swedish island of Gotland, has survived without varroa mite control since 2000, despite consistently high mite infestation rates^35,36^. While this population expresses mite-resistant traits, such as inhibiting the mite’s reproductive success rates^37^, colonies also survive better with the high levels of varroa mite-associated virus infections, which are normally lethal to mite-susceptible bee populations in this region^35^. Individual adult bees of the Gotland mite-resistant (MR) population, compared with those of a mite-susceptible (MS) population, experienced significantly lower mortality rates when inoculated with controlled amounts of ABPV in laboratory cage experiments, even though bees from both populations were equally susceptible to virus infections^38,39^. This work suggests that virus tolerance, rather than virus resistance or reduced susceptibility, is an additional key trait enabling the Gotland honeybee population’s long-term survival under both varroa mite and viral pressure.

This study investigates how mRNA expression levels of key immune-related genes in response to ABPV infection differ between the Gotland MR and local MS honeybee populations, with their contrasting varroa resistance and virus tolerance phenologies. It builds on our previous work showing higher survival rates in adult bees from the Gotland MR population compared to a local MS population under controlled APBV infection^39^. The primary aim is to determine whether immune gene expression patterns differ between ABPV-infected adult bees from these two populations, and to identify at which points during the infection time course such differences are most pronounced. To address this, we analysed the expression profiles of ten genes representing five major immune pathways that have previously been shown to be differentially regulated in relation to ABPV infection^40–42^. By exploring these dynamics, the study aims to provide insight into the molecular mechanisms underlying virus tolerance in honeybees, contributing to a broader understanding of their health and potential for long-term, treatment-free survival in temperate environments.

## Methods

### Experimental design

This study concerns a more detailed analysis of the RNA samples produced by a previous study^39^. The original experimental design consisted of an oral infection time-course of newly emerged adult bees from both the Gotland mite-resistant (MR) population and local mite-susceptible (MS) population that were fed either pre-calibrated amounts of ABPV or buffer (mock inoculation) in a sucrose solution and were subsequently maintained in hoarding cages at constant temperature and humidity. Samples were collected at 12, 24, 48, 72 and 120 h post-inoculation (hpi). Each population was represented by two or three distinct colonies to account for colony-specific effects. Each time course, and its corresponding mock control, was replicated either 3 times (12 and 24 h) or 4 times (48, 72 and 120 h) for each of the five colonies. This resulted in a total of 72 biological samples from five independent bee colonies: three colonies for the MR and two colonies for the MS honeybee populations. RNA was extracted from these 72 samples during autumn and winter 2016^39^. The RNA samples were analysed for the presence of ABPV, to confirm the success of the inoculation protocol. The samples were also assayed for five additional, common, endemic bee viruses whose presence and relative abundance could influence the results (see the Supplementary Material, Appendix 1). The main question addressed with these samples was whether the mRNA expression levels of selected key genes in five major immune gene pathways differed between bees from the two populations in response to ABPV infection, and when during the inoculation time course these differences were most apparent. More precise details of the bee populations, the preparation of the experimental bee colonies, the optimization of the ABPV inoculum, the production of newly emerged adult bees, the logistics of the inoculation time course and sample collection, and the extraction of RNA can be found in Thaduri et al.^39^, and in Appendix 1.

### Selection of immune and internal reference genes for targeted analyses

The strategy for analyzing immune gene expression in these RNA samples involved identifying suitable set of immune and internal reference genes from the literature, then screen the RNA samples for a selection of these genes using RT-qPCR, and compare the changes in the relative expression of these genes between ABPV-inoculated bees relative to mock inoculated bees over time and between the two bee populations.

The most directly comparable immune gene study for our purposes was by Chen et al.^40^, who identified five genes each in four major antiviral immune response signaling cascade pathways (JAK/STAT, mTOR, MAPK and Endocytosis) with expression levels in adult bees strongly influenced by natural IAPV infection. A similar study conducted by DeSmet et al.^41^, yielded a separate set of differentially regulated genes, both in the four above-mentioned immune signaling pathways and in the RNAi pathway, which is the primary molecular defense mechanism in all Eukaryotic organisms against RNA viruses in general^20^. From these two studies we selected two representative genes from each of these five major antiviral pathways to explore the immune response to ABPV infection in adult honeybees from MR colonies, relative to MS colonies. These included: *Dicer-like* (*Dicer*) and *Argonaute-2* (*Ago2*) from the RNA interference (RNAi) pathway; *Dmel* and *GβL* (*GL*) from the mTOR signalling pathway; *Hopscotch* and *Stat* from the JAK/STAT pathway; *Pointed* and *Phi* from the MAPK pathway; and *Past* and *CG1115* from the Endocytosis pathway (Table S1). Detailed descriptions of the genes and their roles in the respective pathways can be found in Brutscher et al.^21^, and Brutscher and Flenniken^22^. Six common honeybee internal reference genes (IRG): *RP49* (a ribosomal protein gene), *GAPDH* (an ubiquitous and abundant metabolic gene), *Amrps18* (*Apis mellifera* 40 S ribosomal protein S18), *Amtif* (*Apis mellifera* translation initiation factor eIF-2B subunit delta), *Amache2* (*Apis mellifera* acetylcholinesterase 2) and *Amtbp* (*Apis mellifera* TATA-box-binding protein), were also included in the analysis, for sample validation and relative quantification analyses^42–44^, as well as qPCR assays for ABPV and five additional common and abundant bee viruses: DWV, *black queen cell virus* (BQCV), *sacbrood virus* (SBV), *lake sinai virus* (LSV) and *chronic bee paralysis virus* (CBPV) whose presence and amounts may influence the data and conclusions^45–48^.

### Confirmation of RNA integrity

Since we were working with RNA samples that were produced in Autumn 2016^39^; Appendix 1), seven years prior to their re-use in the current experiments in Autumn 2023, our first task was to confirm that the condition and integrity of the RNA samples had not degraded significantly since 2016, in order that our current data and results could be compared and analysed in accordance with the original experimental design from 2016. We used two different approaches for this. The first approach was to submit a selection of 15 samples to two different systems specifically designed to detect RNA degradation (Qubit and TapeStation; see Appendix 1 for details). The RNA concentrations determined by these two systems were then compared to the concentrations determined in 2016 using NanoDrop, which does not differentiate between intact and degraded RNA. The results of these analyses are shown in Table S2 and Figure S1. The absolute RNA concentrations in the 15 samples as determined by Qubit and TapeStation in 2023 are similar to those produced by NanoDrop in 2016, with furthermore high positive correlations with the 2016 NanoDrop results (R^2^ = 0.989 for Qubit, R^2^ = 0.875 for TapeStation), neither of which would be expected with significant degradation. For comparison, the correlation between the 2016 and 2023 NanoDrop estimates (which does not account for degradation) was R2 = 0.998, i.e. nearly identical. Also, the RIN profiles produced by the TapeStation were highly acceptable, with most samples registering RIN values between 9 and 10 (Supplementary Figure S2), indicating mostly intact RNA.

The second approach was more functional. We re-ran the RP49, DWV and ABPV assays of 2016^39^ in 2023 with newly produced cDNA and compared the 2023 and 2016 CQ values in a ratio (CQ^2023^/CQ^2016^), for each of the 72 samples. In a perfect world, this ratio should be 1.00. Values above 1.00 indicate lower-than-expected performance, which could be due to RNA degradation. The average CQ^2023^/CQ^2016^ ratio across all 72 samples was slightly above 1.00 for all three assays (1.03 for ABPV, 1.05 for DWV, 1.11 for RP49), with highly uniform figures across all samples for ABPV (Coefficient of Variation; *CV* = 0.053) and RP49 (*CV* = 0.073) and more variable ratios for DWV (*CV* = 0.651).

These chemical and functional analyses confirm that, across the experiment, the integrity of the RNA samples from 2016 was largely conserved over time, with only minimal degradation by 2023, and retained sufficient quality for a trustworthy gene expression analysis using RT-qPCR.

### Confirmation of ABPV inoculation protocol and presence of other viruses

The second task was to confirm with fresh RT-qPCR assays (Table S1) that the 2016 ABPV infection protocol had been successful, as well as to record the presence and amounts of DWV, BQCV and SBV, LSV and CBPV, all of which are common and abundant bee viruses with potential to cause disease and influence both bee mortality and the expression levels of the immune genes^45–51^. Note also that the inoculum was prepared by propagating semi-pure virus isolates in natural bee pupae, and had a final virus composition of 62% ABPV, 22% IAPV, 1% KBV and 15% SBV (see the Supplementary Material). ABPV was detected in 31/36 ABPV inoculated samples and in only one of the mock-inoculated samples (Fig. S2A), with most of the deviations (non-detection in ABPV-inoculated samples, detection in mock-inoculated samples) registered at the end of the infection time course (120 hpi), confirming both the inoculation protocol and allowing clean immune gene expression comparisons between ABPV-inoculated and mock-inoculated bees over most of the time-course. ABPV titres peaked at 48 hpi for both MR and MS population bees, with a slightly steeper infection slope and higher peak for MR bees than MS bees, followed by a slow decline towards 120 hpi for both populations. DWV was detected in every sample, at quite high but uniform levels across the time course and experimental groups (Fig. S2B), while neither BQCV, SBV, LSV or CBPV were detected in any of the samples.

### De novo cDNA synthesis and qPCR assays

While Thaduri et al.^39^, used a OneStep RT-qPCR approach to assay for ABPV, DWV and RP49, here we decided on a two-step strategy, to economize on the RNA considering the large numbers of assays to be run. The concentrations of the selected RNA samples were re-estimated using a NanoDrop spectrophotometer (ThermoFisher Scientific, Waltham, Massachusetts, USA) and diluted to 100 ng/µL, following which cDNA was synthesized from 2 µL (20 ng) total RNA using the High Capacity cDNA Reverse Transcription Kit (ThermoFisher Scientific, Waltham, Massachusetts, USA) following the manufacturer’s protocol for random hexamer-primed cDNA. A working 1/100 dilution cDNA template plate for the qPCR assays was prepared by mixing 2 µL cDNA with 198 µL sterile water.

The cDNA of the genes and viruses targeted by the different qPCR assays was amplified and quantified using the Bio-Rad CFX-Connect thermocycler and SsoFast EvaGreen qPCR reaction reagents and detection chemistry (Bio-Rad, Hercules, California, USA), in 10 µL volumes containing 2 µL of diluted cDNA template and 0.5 µM of forward and reverse gene-specific primers (Table S1). The same thermocycling profile was used for all assays: 95 °C:30 s + 40x[95 °C:5 s − 58 °C:5 s - read] + MeltCurve of 95 °C:60 s + 65 °C:60 s + [65 °C ~ 95 °C @+0.5 °C:5 s - read] + 10 °C:5 min. The assays were run in 96-well plates containing all the samples and the requisite positive and negative controls, with two replicate plates run for each assay. All positive amplifications were verified for correct product identity by visual inspection of their characteristic melting curves before inclusion in the quantitative analysis. The consistency of the qPCR reactions between the two replicates was determined by calculating the ratio of the duplicate Cq values for each sample. The average of these ratios across samples varied between 0.999 and 1.001 for the different assays, testifying to the high level of technical consistency between the duplicate runs of each assay (Table S1).

### Gene expression normalization and relative quantification

The variation in gene expression for the various immune genes targeted was analysed using a relative quantification approach, using the *Cbl* gene mRNA transcript levels to Normalize between all samples (experimental as well as control) for sample-specific differences in RNA quality and quantity regarding RT-qPCR detection and quantification^43,52^. In the current study, the *Cbl* gene was the least variant of all genes investigated in this study across the entire experiment (see the Supplementary Material), and thus most suited for use as an IRG for between sample Normalization of the mRNA levels of the other genes^42^.

The Normalization of the mRNA levels of each target gene by the IRG across all samples was done using the assay efficiency-adjusted formulas developed by Pfaffl^53^; Pfaffl et al., 2004). The relative quantification (RQ) of the mRNA levels of the target gene in response to ABPV infection was subsequently calculated as the ratio of the Normalized mRNA levels in the ABPV-inoculated sample to those of the corresponding mock-inoculated sample^53^; Pfaffl et al., 2004). The various formulas used to calculate these RQ values can be found in Appendix 1.

### Statistical analysis

Linear mixed models were used to analyse the impact of ABPV infection on the RQ values of the 10 immune genes through time in both MR and MS bee colonies, with the population (MR vs. MS), time (12 h, 24 h, 48 h, 72 h and 120 h), and their interaction included as fixed factors, and the colony ID as a random factor. The significances were checked with ANOVA followed by pairwise comparisons of estimated margin means. Five different comparisons were run at each time point as follows: MR population vs. MS population at 12 h, 24 h, 48 h, 72 h and 120 h. P-values were corrected using the Benjamini–Hochberg False Discovery Rate correction^54^.

The data were analysed using R v4.3.3 (R Core Team^55^. Linear mixed modelling was conducted with the ‘lme4’ package (v1.1–35−5); simulation-based diagnostics of mixed model residuals was conducted with the ‘DHARMa’ package (v0.4.7). Packages ‘car’ (v3.1–2; companion package to applied regressions and ANOVA), ‘emmeans’ (v.2.0.0; Pairwise comparisons and False Discovery Rate corrections), ‘performance’ (v0.15.2; evaluate and check statistical models), ‘ggplot2’ (v4.0.0; data visualization), ‘dplyr’ (v1.1.4; data manipulation and data transformation), and ‘patchwork’ (v1.3.2; combine separate plots into the same figure) were used at various points for analysing model performance and producing graphs and figures.

## Results

**Fig. 1:**
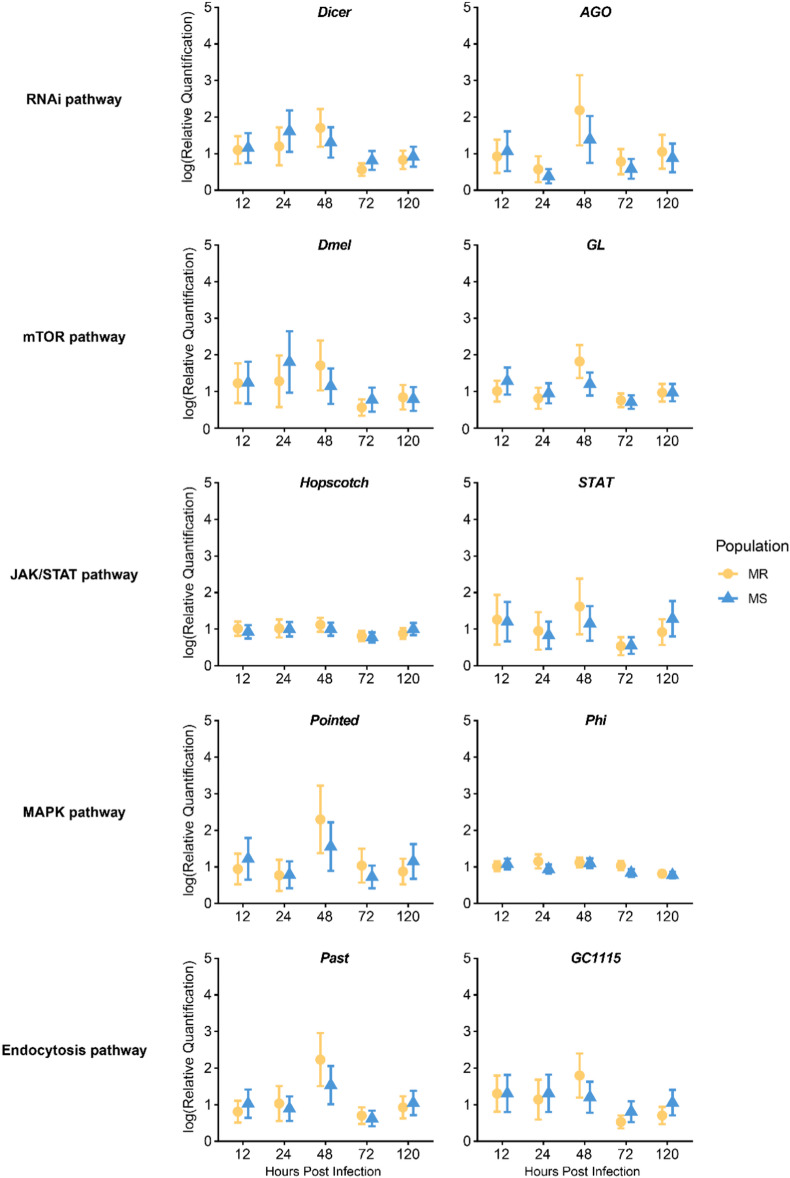
Relative quantification across time of the expression of 10 immune genes in five major immune signalling pathways in response to ABPV infection of adult bees from the Gotland varroa-resistant population (MR, yellow) and adult bees from a varroa-susceptible population (MS, blue).Each data point is based on duplicate RT-qPCR assays for multiple independent infection assays for adult bees from three distinct MR or MS colonies, with the error terms derived from the highest level of replication (i.e. colony level).

The relative quantification (i.e. the ratio of Normalized mRNA levels of a target gene in APBV-inoculated bees relative to those in the corresponding mock-inoculated samples, or RQ value) of the 10 genes investigated had varying temporal dynamics across the time course, with generally the greatest amount of variability in RQ values around the 24 hpi and 48 hpi time points (Fig. 1). Some genes (*e.g. Hopscotch* and *Phi*) showed barely any deviation or variability from neutral (RQ = 1.0) across the time course. Other genes (e.g. *AGO*, *GL*, *STAT*, *Pointed* and *Past*) showed a clear increase in mRNA towards 48 h, with generally slightly higher levels in the MR bees compared to the MS bees, although this difference was not statistically significant.

Two genes, from two distinct pathways, *Dicer* (RNAi pathway) and *Dmel* (mTOR pathway) exhibited distinct temporal patterns in RQ values between MR and MS bees. In MS bees the RQ values peaked at 24 h followed by a subsequent decline in expression. For MR bees, the RQ expression levels peaked rather at 48 h, before subsequently declining. While these temporal differences were observed in the data, they were not statistically significant in the various analytical models (Tables S3, S4, S5).

## Discussion

This study investigated the effects of oral ABPV inoculation of newly emerged adult honeybees from varroa-resistant and varroa-susceptible populations^39^ on the relative expression levels of 10 immune genes in five major immune pathways (JAK/STAT, mTOR, MAPK, Endocytosis and RNAi) previously shown to be highly responsive to IAPV/ABPV infection^40,41^. We identified temporal ABPV-inoculation associated increases in mRNA levels for many, but not all, of the immune genes investigated, with uniquely distinct responses to the ABPV treatment between genes, including several that showed no response at all. For those genes that did respond to ABPV infection, there was generally a peak in elevated mRNA levels around 24 to 48 hpi, followed by a decline back to neutral, with generally 1.5 to 2-fold increase in mRNA levels in ABPV-inoculated bees relative to mock-inoculated bees. There were also minor (and statistically non-significant) differences between adult bees from the MR and MS populations in how their immune systems, as represented by the selected genes, responded to ABPV infection, with mRNA levels in MR bees peaking slightly later, and higher, than in MS bees. There was slight evidence for a possible clearance of ABPV infection with time, with a small number of late-stage (120 hpi) ABPV-inoculated samples no longer having detectable levels of ABPV. That bees can recover from ABPV infection over time is supported by a recent independent study that showed progressively lower virus levels for ABPV-infected bees, but not for DWV infected bees^50^. Despite clear temporal effects for some genes, as well as unique and consistent differences between genes, across the study there was uniformly a lack of statistical significance for many effects, particularly for differences between the populations. This likely reflects the limit in the power of the fitted models. While the sample size (*n* = 72) is typical for molecular studies, and adequate in general, it was partitioned across many different factors (population, treatment, time) and their interactions, reducing the ability to assign statistical significance to more subtle effects. One obvious limitation of the current study is therefore the relatively paucity of colony-level replication, which coincidentally also often harbours the greatest amount of biological variation, in all types of honeybee research. This meant that the study was intrinsically rather underpowered for answering population-level questions, while still being sufficient for the ABPV inoculation and time-course questions, and this was largely reflected in our results and conclusions.

The difference in how these genes responded to ABPV infection in our study compared to the previous studies^40–42^ is also not entirely unexpected. Apart from the radically different technologies used to detect and analyse the changes in immune gene expression, there were also major differences in the experimental design, with both^40,41^ analysing rather poorly resolved, naturally infected, colony-level samples, while our current study had a more experimental structure, with much greater control over infection conditions and bee age and origins. The three previous studies also differed considerably among each other in which immune genes responded most to the presence of ABPV/IAPV, despite having much more similar experimental designs than the current study. Another factor that could have contributed to the differences between these studies is the relative and absolute levels of other pathogens, which would be unique and different for each study. In our study we confirmed a considerable background of DWV, whose amounts were equitably distributed over the samples and whose levels were taken into account in the experimental design, while four other viruses were assayed for, but not detected. Such variable results from comparable, but not identical, studies are rather common for gene expression studies in general, and reflects both the dynamic, interactive and unpredictable nature of the innate immune response, compounded by methodological, analytical and experimental design differences, as well as biases inherent in gene expression analysis in general.

In this study we screened RNA samples from an extensive infection time-course experiment using a targeted panel of representative genes from the major immune gene pathways by RT-qPCR. This approach confirmed the quality of the RNA for downstream gene expression analyses and provided insight into temporal and gene-specific responsiveness patterns of the honey bee immune system to a viral challenge. While differential response of the target genes between bees from the two populations was not observed here, our ambition is to extend this work with more comprehensive gene expression technologies, such as RNA sequencing, allowing for the interactive mapping of entire gene expression pathways and enable a systems-level understanding of the regulatory networks that underpin variation in antiviral defence to help clarify the mechanisms driving differences between bee populations.

## Supplementary Information

Below is the link to the electronic supplementary material.

Supplementary Material 1

## Data Availability

The datasets generated during the current study are available from the corresponding author on reasonable request.
