# Supplementary material for: Experimental infection of acute bee paralysis virus induces differential responses of selected honey bee immune genes, independent of varroa resistance
Source: Sci Rep. 2026 Aug 5;16:24199. doi: 10.1038/s41598-026-65201-4 (PMC13442843; doi:10.1038/s41598-026-65201-4)
Supplement: Supplementary file 1 [file 41598_2026_65201_MOESM1_ESM.docx]

**Supplementary Material**

**Experimental infection of *acute bee paralysis virus* induces differential responses of selected honey bee immune genes, independent of varroa resistance**

**Xinyan RUAN^1^*, Amélie NOËL^1^, Srinivas THADURI^2^, Joachim R. DE MIRANDA^1^, Barbara LOCKE^1^**

^1^ Honey Bee Research Centre, Department of Ecology, Swedish University of Agricultural Sciences, Uppsala, 756 51, Sweden

^2^ Surabhi Institute of Medical Science, Siddipet, Telananga, 502375, India

*Corresponding author: Xinyan RUAN: [xinyan.ruan@slu.se](mailto:xinyan.ruan@slu.se)

Amélie NOËL: amelie.noel@slu.se

Srinivas THADURI: srinivas.thaduri2801@gmail.com

Joachim R. DE MIRANDA: joachim.de.miranda@slu.se

Barbara LOCKE: barbara.locke@slu.se

**Figures and Tables**

**Figure S1.** Confirmation of RNA integrity.

**Figure S2.** Confirmation of ABPV inoculation protocol and virus detection.

**Table S1.** RT-qPCR assays.

**Table S2.** Confirmation of RNA integrity.

**Table S3.** ANOVA of the population and time effects on the relative quantification.

**Table S4.** Pairwise comparison of the Treatment effects within each population on the expression levels of the immunity genes.

**Table S5.** Pairwise comparison results to showed population and time effects on the relative quantification

**Figure S1.** Agilent RNA ScreenTape analysis

***Legend:*** ScreenTape analysis of 15 random RNA samples from the 2016 virus inoculation experiments (Thaduri *et al.,* 2019). Shown is a ladder of size standards (lane 1), the original LabIDs of the samples and their corresponding RIN values as calculated by the TapeStation software. LabIDs in black font are included in the current experiment (see data file). LabIDs in grey font derive from other parts of the 2016 experiment, such as adult bees inoculated with DWV (083, 158), larval samples inoculated with DWV (291) or not (290), and adult samples inoculated with ABPV that were either alive (082) or dead (126) when collected.

**Figure S2.** Confirmation ABPV inoculation protocol and virus quantification

***Legend:*** Re-calculated absolute levels of ABPV and DWV in the RNA samples from the ABPV-inoculated (dark colours) and mock-inoculated (light colours) adult bees from the MR (yellow) and MS (blue) bee population colonies, as resolved by the time course. Values are given in estimated genome copy numbers of each virus, on a Log_10_ scale. The graphs show raw mean values with simple standard deviations based on the number of samples in each category.

**Table S1.** RT-qPCR assays

***Legend:*** Primer sequences and qPCR performance parameters for the qPCR assays used in this study. Shown is the assay name, its pathway, the name and sequence of each primer, the GenBank accession number of the gene targeted, the size of the PCR product, the assay amplification efficiency (mean and standard deviation), the linearity (R^2^) of the log-linear relationship between Cq value and the amount of target gene, as determined from the external dilution standards series, the Coefficient of Variation (CV) of the ratio of the duplicate Cq values for each assay, the average Cq value for the assay across all 72 samples as well as the average CV value for the mean and standard deviation of the duplicate Cq values, and finally the original reference for each of the assays.

**Table S2.** RNA stability metrics

***Legend:*** Summary of the chemical stability metrics for a selection of the 2016 RNA samples, as determined by NanoDrop, Cubit and TapeStation. Shown are the original sample Lab_ID, the ID of the bee colony the sample came from, the bee population this colony belonged to (MR or MS), the life-stage of the bee (adult, larva), the inoculum (ABPV, DWV, mock) fed to the bees, the life status (alive, dead) of the bee upon collection, the collection date, the stage in the time course the sample came from, the NanoDrop values (concentration in ng/ul, 260/280 ratio and 260/230 ratio) from 2016 and 2023, the concentration as determined by Cubit and TapeStation (both in 2023), as well as the TapeStation RIN and RNAIQ values, both on a scale 1-10 with high values indicating less degradation. Also shown are the correlations (R^2^) between the NanoDrop RNA concentrations of 2016 and 2023, as well as between the 2023 NanoDrop concentration and the 2023 Cubit or TapeStation concentrations. All samples used in this study originated from the previous study by Thaduri et al. (2019), and only a subset of the original samples was included in the present analyses. The samples shaded in dark grey were included among the 72 samples of the current study.

**Table S3.** ANOVA of the population and time effects on the relative quantification.

| Gene | Pathway | Population | | | Time | | | Population*Time | | |
| --- | --- | --- | --- | --- | --- | --- | --- | --- | --- | --- |
|  |  | df | chisq | P value | df | chisq | P value | df | Chisq | P value |
| *Dicer* | RNAi | 1 | 0.23 | 0.63 | 4 | 9.30 | 0.054 | 4 | 1.31 | 0.86 |
| *AGO* | RNAi | 1 | 0.45 | 0.50 | 4 | 0.62 | **0.047** | 4 | 0.53 | 0.97 |
| *GL* | mTOR | 1 | 0.08 | 0.78 | 4 | 9.06 | 0.06 | 4 | 1.89 | 0.76 |
| *Dmel* | mTOR | 1 | 0.0025 | 0.96 | 4 | 7.49 | 0.11 | 4 | 1.33 | 0.86 |
| *Past* | Endosynthesis | 1 | 0.08 | 0.77 | 4 | 11.08 | **0.02** | 4 | 1.02 | 0.91 |
| *GC1115* | Endosynthesis | 1 | 0.28 | 0.60 | 4 | 7.84 | 0.097 | 4 | 2.10 | 0.72 |
| *Phi* | MAPK | 1 | 1.15 | 0.28 | 4 | 10.25 | **0.036** | 4 | 1.79 | 0.77 |
| *Pointed* | MAPK | 1 | 0.02 | 0.90 | 4 | 6.68 | 0.15 | 4 | 1.44 | 0.84 |
| *Hopscotch* | JAK/STAT | 1 | 0.05 | 0.81 | 4 | 3.35 | 0.50 | 4 | 0.63 | 0.96 |
| *STAT* | JAK/STAT | 1 | 0.001 | 0.97 | 4 | 6.18 | 0.19 | 4 | 0.79 | 0.94 |

***Legend:*** Results of linear mixed-effects models (LMMs) analysing the effects of ABPV infection on relative quantification (RQ) of 10 immune genes over time in MR and MS colonies. Population (MR *vs.* MS), time (12 h, 24 h, 48 h, 72 h, 120 h), and their interaction were included as fixed effects, with colony ID as a random effect. The table shows the degrees of freedom (df), χ² values, and P values for population, time, and their interaction. Bold indicates P < 0.05 (suggesting that further pairwise comparisons are warranted).

**Table S4.** Pairwise comparison of the Treatment effects within each population on the expression levels of the immunity genes.

| **Gene** | **Pathway** | **Time** | **Population** | **ABPV (mean ± SE)** | **Sugar (mean ± SE)** | **p-value** |
| --- | --- | --- | --- | --- | --- | --- |
| *Dicer* | RNAi | 12h | MR | 1.34 ± 0.13 | 1.21 ± 0.13 | 0.29 |
|  |  |  | MS | 1.33 ± 0.15 | 1.15 ± 0.15 | 0.15 |
|  |  | 24h | MR | 0.96 ± 0.11 | 0.87 ± 0.14 | 0.60 |
|  |  |  | MS | 1.31 ± 0.12 | 0.81 ± 0.12 | **0.02** |
|  |  | 48h | MR | 1.50 ± 0.34 | 0.94 ± 0.34 | 0.25 |
|  |  |  | MS | 1.21 ± 0.37 | 0.90 ± 0.37 | 0.52 |
|  |  | 72h | MR | 0.92 ± 0.28 | 1.51 ± 0.28 | 0.056 |
|  |  |  | MS | 1.23 ± 0.32 | 1.59 ± 0.32 | 0.21 |
|  |  | 120h | MR | 1.04 ± 0.22 | 1.19 ± 0.22 | 0.50 |
|  |  |  | MS | 0.70 ± 0.24 | 0.85 ± 0.25 | 0.54 |
| *AGO* | RNAi | 12h | MR | 7.15 ± 1.49 | 7.72 ± 1.60 | 0.80 |
|  |  |  | MS | 5.96 ± 1.35 | 5.66 ± 1.28 | 0.87 |
|  |  | 24h | MR | 2.18 ± 0.32 | 2.42 ± 0.42 | 0.64 |
|  |  |  | MS | 1.57 ± 0.36 | 2.54 ± 0.36 | 0.07 |
|  |  | 48h | MR | 6.92 ± 3.82 | 3.19 ± 1.76 | 0.30 |
|  |  |  | MS | 6.38 ± 3.88 | 4.68 ± 2.85 | 0.67 |
|  |  | 72h | MR | 4.01 ± 1.12 | 5.14 ± 1.43 | 0.52 |
|  |  |  | MS | 6.14 ± 1.88 | 10.69 ± 3.28 | 0.17 |
|  |  | 120h | MR | 8.84 ± 1.91 | 8.47 ± 1.83 | 0.89 |
|  |  |  | MS | 10.12 ± 2.12 | 11.58 ± 2.77 | 0.67 |
| *Dmel* | mTOR | 12h | MR | 1.54 ± 0.18 | 1.24 ± 0.18 | 0.29 |
|  |  |  | MS | 1.41± 0.19 | 1.19 ± 0.19 | 0.42 |
|  |  | 24h | MR | 0.81 ± 0.18 | 0.81 ± 0.24 | 0.99 |
|  |  |  | MS | 1.46 ± 0.20 | 0.82 ± 0.20 | 0.057 |
|  |  | 48h | MR | 1.30 ± 0.23 | 0.99 ± 0.23 | 0.32 |
|  |  |  | MS | 1.09 ± 0.25 | 0.95 ± 0.25 | 0.64 |
|  |  | 72h | MR | 0.94 ± 0.32 | 1.34 ± 0.32 | 0.37 |
|  |  |  | MS | 1.10 ± 0.35 | 1.51 ± 0.35 | 0.36 |
|  |  | 120h | MR | 1.16 ± 0.28 | 1.47 ± 0.28 | 0.33 |
|  |  |  | MS | 1.06 ± 0.31 | 1.26 ± 0.32 | 0.55 |
| *GL* | mTOR | 12h | MR | 1.53 ± 0.12 | 1.52 ± 0.12 | 0.96 |
|  |  |  | MS | 1.49 ± 0.13 | 1.16 ± 0.13 | 0.11 |
|  |  | 24h | MR | 1.21 ± 0.30 | 1.30 ± 0.39 | 0.86 |
|  |  |  | MS | 1.39 ± 0.33 | 1.50 ± 0.33 | 0.64 |
|  |  | 48h | MR | 1.41 ± 0.27 | 0.96 ± 0.27 | 0.13 |
|  |  |  | MS | 1.19 ± 0.30 | 1.08 ± 0.30 | 0.71 |
|  |  | 72h | MR | 1.16 ± 0.20 | 1.36 ± 0.20 | 0.42 |
|  |  |  | MS | 1.16 ± 0.22 | 1.63 ± 0.22 | 0.08 |
|  |  | 120h | MR | 1.26 ± 0.13 | 1.28 ± 0.13 | 0.93 |
|  |  |  | MS | 1.45 ± 0.12 | 1.50 ± 0.14 | 0.79 |
| *Past* | Endosynthesis | 12h | MR | 4.16 ± 0.79 | 5.11 ± 0.79 | 0.43 |
|  |  |  | MS | 5.06 ± 0.86 | 4.99 ± 0.86 | 0.95 |
|  |  | 24h | MR | 4.86 ± 0.94 | 4.27 ± 1.23 | 0.68 |
|  |  |  | MS | 4.70 ± 1.05 | 5.46 ± 1.05 | 0.53 |
|  |  | 48h | MR | 5.38 ± 0.85 | 3.23 ± 0.85 | 0.09 |
|  |  |  | MS | 4.62 ± 0.94 | 3.50 ± 0.94 | 0.35 |
|  |  | 72h | MR | 3.52 ± 0.60 | 4.37 ± 0.60 | 0.24 |
|  |  |  | MS | 3.87 ± 0.68 | 6.18 ± 0.68 | **0.007** |
|  |  | 120h | MR | 3.43 ± 0.86 | 3.52 ± 0.86 | 0.94 |
|  |  |  | MS | 3.72 ± 0.87 | 3.52 ± 0.95 | 0.86 |
| *GC1115* | Endosynthesis | 12h | MR | 3.27 ± 0.52 | 2.70 ± 0.52 | 0.43 |
|  |  |  | MS | 4.22 ± 0.57 | 3.19 ± 0.57 | 0.18 |
|  |  | 24h | MR | 1.89 ± 0.29 | 1.78 ± 0.38 | 0.82 |
|  |  |  | MS | 2.93 ± 0.32 | 2.24 ± 0.32 | 0.16 |
|  |  | 48h | MR | 2.95 ± 0.68 | 1.98 ± 0.68 | 0.16 |
|  |  |  | MS | 2.20 ± 0.78 | 1.94 ± 0.78 | 0.68 |
|  |  | 72h | MR | 1.73 ± 0.47 | 2.41 ± 0.47 | 0.30 |
|  |  |  | MS | 2.53 ± 0.52 | 3.25 ± 0.52 | 0.28 |
|  |  | 120h | MR | 1.98 ± 0.60 | 2.82 ± 0.60 | 0.25 |
|  |  |  | MS | 2.58 ± 0.63 | 2.55 ± 0.67 | 0.98 |
| *Phi* | MAPK | 12h | MR | 1.30 ± 0.08 | 1.28 ± 0.08 | 0.82 |
|  |  |  | MS | 1.31 ± 0.09 | 1.21 ± 0.08 | 0.32 |
|  |  | 24h | MR | 1.14 ± 0.14 | 0.92 ± 0.15 | 0.34 |
|  |  |  | MS | 1.13 ± 0.15 | 1.20 ± 0.16 | 0.73 |
|  |  | 48h | MR | 1.18 ± 0.10 | 1.06 ± 0.09 | 0.28 |
|  |  |  | MS | 1.07 ± 0.10 | 0.97 ± 0.09 | 0.35 |
|  |  | 72h | MR | 1.18 ± 0.10 | 1.13 ± 0.09 | 0.73 |
|  |  |  | MS | 1.15 ± 0.11 | 1.38 ± 0.13 | 0.14 |
|  |  | 120h | MR | 1.01 ± 0.11 | 1.24 ± 0.14 | 0.15 |
|  |  |  | MS | 0.89 ± 0.10 | 1.12 ± 0.14 | 0.13 |
| *Pointed* | MAPK | 12h | MR | 2.14 ± 0.27 | 2.20 ± 0.27 | 0.89 |
|  |  |  | MS | 2.24 ± 0.29 | 2.00 ± 0.29 | 0.54 |
|  |  | 24h | MR | 1.76 ± 0.29 | 1.99 ± 0.38 | 0.60 |
|  |  |  | MS | 1.64 ± 0.33 | 2.34 ± 0.33 | 0.102 |
|  |  | 48h | MR | 2.26 ± 0.61 | 1.47 ± 0.61 | 0.34 |
|  |  |  | MS | 1.93 ± 0.67 | 2.09 ± 0.67 | 0.85 |
|  |  | 72h | MR | 1.94 ± 0.20 | 1.87 ± 0.19 | 0.80 |
|  |  |  | MS | 1.88 ± 0.21 | 2.81 ± 0.21 | **0.0055** |
|  |  | 120h | MR | 2.04 ± 0.46 | 2.36 ± 0.46 | 0.56 |
|  |  |  | MS | 2.20 ± 0.47 | 2.15 ± 0.51 | 0.93 |
| *Hopscotch* | JAK/STAT | 12h | MR | 0.62 ± 0.06 | 0.61 ± 0.06 | 0.89 |
|  |  |  | MS | 0.56 ± 0.06 | 0.60 ± 0.06 | 0.58 |
|  |  | 24h | MR | 0.53 ± 0.04 | 0.52 ± 0.06 | 0.89 |
|  |  |  | MS | 0.56 ± 0.05 | 0.56 ± 0.05 | 1.00 |
|  |  | 48h | MR | 0.61 ± 0.08 | 0.55 ± 0.08 | 0.59 |
|  |  |  | MS | 0.49 ± 0.09 | 0.52 ± 0.09 | 0.76 |
|  |  | 72h | MR | 0.51 ± 0.10 | 0.61 ± 0.10 | 0.40 |
|  |  |  | MS | 0.62 ± 0.11 | 0.83 ± 0.11 | 0.10 |
|  |  | 120h | MR | 0.47 ± 0.08 | 0.52 ± 0.08 | 0.67 |
|  |  |  | MS | 0.51 ± 0.08 | 0.50 ± 0.09 | 0.92 |
| *STAT* | JAK/STAT | 12h | MR | 2.76 ± 0.93 | 4.98 ± 0.93 | 0.15 |
|  |  |  | MS | 5.22 ± 1.02 | 4.50 ± 1.02 | 0.61 |
|  |  | 24h | MR | 4.60 ± 1.00 | 4.80 ± 1.00 | 0.84 |
|  |  |  | MS | 2.77 ± 1.14 | 5.46 ± 1.14 | **0.037** |
|  |  | 48h | MR | 3.90 ± 0.81 | 4.31 ± 0.81 | 0.70 |
|  |  |  | MS | 4.96 ± 0.94 | 4.28 ± 0.89 | 0.56 |
|  |  | 72h | MR | 4.88 ± 1.43 | 3.68 ± 1.43 | 0.49 |
|  |  |  | MS | 4.43 ± 1.63 | 3.51 ± 2.15 | 0.64 |
|  |  | 120h | MR | 3.69 ± 0.79 | 4.75 ± 0.79 | 0.32 |
|  |  |  | MS | 5.78 ± 0.79 | 5.65 ± 0.92 | 0.91 |

***Legend:*** Pairwise comparison results showing the effects of treatment within each population on the expression levels of immune genes following ABPV infection. Estimated marginal means (mean ± standard error, SE, 95% confidence intervals) are presented for ABPV-infected and control (sugar only) groups within each population (MR and MS) at each time point (12 h, 24 h, 48 h, 72 h, 120 h). P-values indicate the significance of differences between treatments within each population and time point. Bold indicates P < 0.05 (suggesting that there is significant difference between treatment within this population at that time point).

**Table S5.** Pairwise comparison results to showed population and time effects on the relative quantification

| Gene | Pathway | Time | MR (response ± se) | MS (response ± se) | P value |
| --- | --- | --- | --- | --- | --- |
| *Dicer* | RNAi | 12 | 1.10 ± 0.38 | 1.16 ± 0.40 | 0.92 |
|  |  | 24 | 1.20 ± 0.52 | 1.62 ± 0.57 | 0.60 |
|  |  | 48 | 1.70 ± 0.52 | 1.31 ± 0.42 | 0.55 |
|  |  | 72 | 0.56 ± 1.17 | 0.82 ± 0.26 | 0.41 |
|  |  | 120 | 0.83 ± 0.25 | 0.92 ± 0.27 | 0.82 |
| *AGO* | RNAi | 12 | 0.93 ± 0.46 | 1.06 ± 0.54 | 0.85 |
|  |  | 24 | 0.57 ± 0.35 | 0.38 ± 0.20 | 0.62 |
|  |  | 48 | 2.19 ± 0.96 | 1.39 ± 0.64 | 0.49 |
|  |  | 72 | 0.78 ± 0.34 | 0.59 ± 0.27 | 0.66 |
|  |  | 120 | 1.05 ± 0.46 | 0.88 ± 0.39 | 0.78 |
| *Dmel* | mTOR | 12 | 1.22 ± 0.54 | 1.24 ± 0.58 | 0.99 |
|  |  | 24 | 1.28 ± 0.70 | 1.80 ± 0.84 | 0.64 |
|  |  | 48 | 1.71 ± 0.68 | 1.14 ± 0.48 | 0.50 |
|  |  | 72 | 0.56 ± 0.22 | 0.78 ± 0.33 | 0.58 |
|  |  | 120 | 0.84 ± 0.34 | 0.79 ± 0.32 | 0.92 |
| *GL* | mTOR | 12 | 1.00 ± 0.28 | 1.28 ± 0.37 | 0.55 |
|  |  | 24 | 0.81 ± 0.29 | 0.95 ± 0.27 | 0.74 |
|  |  | 48 | 1.82 ± 0.45 | 1.20 ± 0.31 | 0.26 |
|  |  | 72 | 0.76 ± 0.19 | 0.71 ± 0.18 | 0.86 |
|  |  | 120 | 0.96 ± 0.24 | 0.97 ± 0.24 | 0.99 |
| *Pointed* | MAPK | 12 | 0.94 ± 0.42 | 1.22 ± 0.57 | 0.69 |
|  |  | 24 | 0.77 ± 0.43 | 0.78 ± 0.37 | 0.98 |
|  |  | 48 | 2.30 ± 0.92 | 1.56 ± 0.66 | 0.52 |
|  |  | 72 | 1.04 ± 0.46 | 0.73 ± 0.31 | 0.58 |
|  |  | 120 | 0.87 ± 0.35 | 1.15 ± 0.47 | 0.64 |
| *Phi* | MAPK | 12 | 1.01 ± 0.13 | 1.08 ± 0.14 | 0.76 |
|  |  | 24 | 1.15 ± 0.19 | 0.94 ± 0.12 | 0.35 |
|  |  | 48 | 1.12 ± 0.13 | 1.10 ± 0.13 | 0.92 |
|  |  | 72 | 1.04 ± 0.12 | 0.84 ± 0.10 | 0.22 |
|  |  | 120 | 0.81 ± 0.09 | 0.78 ± 0.09 | 0.77 |
| *Past* | Endosynthesis | 12 | 0.81 ± 0.30 | 1.03 ± 0.39 | 0.66 |
|  |  | 24 | 1.03 ± 0.48 | 0.89 ± 0.34 | 0.81 |
|  |  | 48 | 2.23 ± 0.72 | 1.53 ± 0.52 | 0.44 |
|  |  | 72 | 0.70 ± 0.23 | 0.62 ± 0.21 | 0.81 |
|  |  | 120 | 0.92 ± 0.30 | 1.04 ± 0.33 | 0.80 |
| *GC1115* | Endosynthesis | 12 | 1.30 ± 0.50 | 1.30 ± 0.51 | 0.99 |
|  |  | 24 | 1.14 ± 0.54 | 1.31 ± 0.51 | 0.82 |
|  |  | 48 | 1.80 ± 0.60 | 1.20 ± 0.42 | 0.42 |
|  |  | 72 | 0.53 ± 0.18 | 0.81 ± 0.28 | 0.39 |
|  |  | 120 | 0.70 ± 0.24 | 1.06 ± 0.35 | 0.40 |
| *Hopscotch* | JAK/STAT | 12 | 1.01 ± 0.20 | 0.93 ± 0.18 | 0.75 |
|  |  | 24 | 1.02 ± 0.25 | 1.00 ± 0.20 | 0.95 |
|  |  | 48 | 1.12 ± 0.19 | 1.00 ± 0.18 | 0.65 |
|  |  | 72 | 0.81 ± 0.14 | 0.77 ± 0.14 | 0.84 |
|  |  | 120 | 0.88 ± 0.15 | 1.00 ± 0.17 | 0.60 |
| *STAT* | JAK/STAT | 12 | 1.60 ± 0.68 | 1.20 ± 0.54 | 0.95 |
|  |  | 24 | 0.95 ± 0.51 | 0.83 ± 0.37 | 0.85 |
|  |  | 48 | 1.62 ± 0.76 | 1.16 ± 0.47 | 0.60 |
|  |  | 72 | 0.53 ± 0.24 | 0.55 ± 0.23 | 0.96 |
|  |  | 120 | 0.92 ± 0.35 | 1.28 ± 0.48 | 0.54 |

***Legend:*** Pairwise comparison results showing the effects of population and time on the relative quantification (RQ) of immune gene expression following ABPV infection. Estimated marginal means (response ± standard error, SE, 95% confidence intervals) are presented for MR and MS populations at each time point (12 h, 24 h, 48 h, 72 h, 120 h). P-values indicate the significance of differences between populations within each time point.

**Appendix 1 – Detailed Methods**

**Experimental Design**

The basic experimental design of the original study (Thaduri *et al.,* 2019) was an infection time-course, with samples taken at 12, 24, 48, 72 and 120 hours post-inoculation. Separate infection time-course experiments were conducted for three types of inocula (ABPV, DWV and a virus-free mock inoculum), for two life-stages (larvae and newly emerged adult worker bees) and for two different bee population origins (the 25+ year old varroa-resistant population on Gotland and a local varroa-susceptible honey bee population), with various degrees of replication at all stages of the experiment. RNA was extracted from the collected samples to quantify the levels of ABPV, DWV and internal reference genes. These RNA samples form the basis for the current study. We selected 78 RNA samples from the original study for immune gene analyses, using the following criteria:

1. Adult bee samples – Only samples from the adult bee study were included, since these provided the most interesting associated phenotypic data (longevity and survival) and included all five sampling time-points, for optimal time-course resolution of expression level changes for a diverse range of target genes.
2. ABPV inoculum – Only the ABPV and mock inoculations were included, since only ABPV showed both proven infectivity and no background infection in uninoculated bees, i.e. the cleanest possible infection experiment.
3. Surviving bees – Only surviving bees were included, since these produced the most reliable RNA and the most interesting associated phenotypes.
4. Colonies – Only colonies with both ABPV and mock inoculation time-courses were included, to account for colony-specific effects.
5. Populations – Each population was represented by two independent colonies.

**Honey bee material**

The experimental bee colonies were prepared during summer 2016 by splitting bee colonies imported from Åland, an island archipelago between Sweden and Finland with a bee population that, at the time of the experiments, was completely naïve to both varroa and its viruses (Doublet *et al.,* 2024) and re-queening each split with a newly open-mated queen from either the local Uppsala varroa-susceptible bee population (MS colonies) or from the varroa-resistant population on Gotland (MR colonies). The colonies were thus varroa- and virus-free at the start of the experiment, with the only difference the origin of the queen. The colonies were established in 6-frame hives in a separate apiary at SLU in Uppsala during May-June 2016. Experiments began two months after re-queening, to ensure that all bees in the colony were offspring of the introduced queen (Thaduri *et al.,* 2019).

**Virus material**

The ABPV used in the oral infection experiment was propagated by injecting 50 white-eyed pupae from Åland colonies with 1 μLof a 1/10,000 dilution of purified concentrated virus stock (equivalent to about 1000 genome copies per bee), according to standard pupal propagation procedures (De Miranda et al., 2013). Crude extracts were stored at -80°C. Infectivity was optimized by identifying the highest virus dose not causing mortality before 96 hours post-inoculation (hpi), enabling assessment of both early infection dynamics during the 120 hour infection time-course window, while retaining sufficient bees for monitoring survival beyond this window. The optimum ABPV dose for adult bees for this purpose was determined by RT-qPCR to be ~2.1× 10^8^ ABPV genome equivalents (Thaduri *et al.,* 2019). Note that such serial propagation of live virus inoculum in natural bee material (pupae) is inherently susceptible to co-propagation of other viruses, either those in the original inoculum or those present in the bee pupae (de Miranda *et al.,* 2013). For this reason, the final virus extracts used for the oral inoculation experiments were tested for the presence and relative abundance of other viruses, and only the purest inoculum was selected for the oral inoculation experiments, which in this case consisted of 62% ABPV, 22% IAPV, 15% SBV and 1% KBV (Thaduri *et al.,* 2019; Figure S3).

**Adult honey bee oral infection experiments**

Multiple cohorts of fifty newly-emerged adult bees from each of three MR and two MS colonies were placed in sterile, plastic 45x50x75 mm^3^ Lyson queen storage cages (Łysoń Beekeeping Company, Klecza Dolna, Poland)), with matching 50 bee cohorts from each colony allocated for either APBV infection or a mock (sugar solution) inoculation. Virus inoculation was performed by bulk-feeding each cohort of 50 bees for 24 hours with virus-spiked Bifor® (Nordic Sugar A/S, Copenhagen, Denmark), followed by chasing the infection with uncontaminated Bifor® for the remainder of the experiment, to ensure that only newly established infections are identified in the time-course samples, with minimal background contribution by the original inoculum. Control groups received virus-free Bifor® throughout the experiment. From each cage trial, 5 live adult bees were sampled at 12, 24, 48, 72 and 120 hours post inoculation and stored at -20°C. The survival rate of each cohort of bees was recorded throughout the time-course, adjusted for the removal of dead and sampled bees (Thaduri *et al.,* 2019).

**RNA extraction**

RNA was extracted from the experimental samples during Autumn-Winter 2016. The samples of 5 adult bees from each cage trial at each time point were placed in a mesh bag and ground into a powder using liquid nitrogen and a pestle. A primary homogenate was prepared by adding 200 µL of sterile water per bee to each ground sample and mixing thoroughly (Thaduri *et al.,* 2019). Total RNA was extracted from 100 µL of the homogenate using a QiaCube robot according to the RNAeasy protocol for plant tissue (Qiagen, Hilden, Germany). The RNA was eluted in 50 µL of RNase-free water, and its concentration and quality were assessed using a NanoDrop spectrophotometer (ThermoFisher Scientific, Waltham, Massachusetts, USA), after which the RNA samples were stored at -80^o^C until further use. This occurred first during spring 2017 for DWV and ABPV quantification (Thaduri *et al.,* 2019). The second usage of these samples was during Autumn 2023, for the current experiments.

**Selection of immune and internal reference genes for targeted analyses**

The experimental approach chosen for immune gene expression analyses for these particular RNA samples was to identify a set of suitable immune and internal reference genes from the literature, screen the RNA samples for these genes using RT-qPCR, and compare the changes in the relative expression of these genes in ABPV inoculated bees relative to mock inoculated bees over time and between the two bee populations. The most directly comparable immune gene study for our purposes was conducted by Chen *et al.,* (2014) who, through microarray screening and RT-qPCR confirmation, identified five genes each in four major antiviral immune response signaling cascade pathways (JAK-STAT, mTOR, MAPK and Endocytosis) whose expression levels in adult bees were most strongly influenced by natural IAPV infection. A similar study conducted by DeSmet *et al.,* (2017) but involving general colony-level virus infections in adult bees, screened with the locally designed BeeClinic microarray loaded with immune genes from the Beepath qPCR array (Evans, 2006) and similarly confirmed by RT-qPCR, yielded a separate set of differentially regulated genes, both in the four above-mentioned immune signaling pathways and in the RNAi pathway, whose function is the sequence-specific digestion of excess mRNA which also makes it the primary molecular defense mechanism in all Eukaryotic organisms against RNA viruses in general (DeSmet *et al.,* 2017; Haasnoot *et al.,* 2007). From these two studies we selected two representative genes from each of these five major antiviral pathways to explore the immune response of ABPV tolerant adult honey bees from varroa-resistant bee colonies, relative to those non-resistant control colonies (Table S1). Six common honey bee internal reference genes: RP49 (a ribosomal protein gene), GAPDH (a ubiquitous and abundant metabolic gene), Amrps18 (*Apis mellifera* 40S ribosomal protein S18), Amtif (*Apis mellifera* translation initiation factor eIF-2B subunit delta), Amache2 (*Apis mellifera* acetylcholinesterase 2) and Amtbp (*Apis mellifera* TATA-box-binding protein), were also included in the analysis, for sample validation and relative quantification analyses (Bustin *et al.,* 2009, Kozera & Rapacz, 2013; Deng *et al.,* 2020), as well as qPCR assays for ABPV and three common and abundant bee viruses (*deformed wing virus*, *black queen cell virus* and *sacbrood virus*), whose presence and amounts may influence the data and conclusions (Table S1). All assays were designed for the specific detection of the mRNA of each gene, generally by locating the forward and reverse primers on different exons of the gene, rendering the genomic version of the amplicon (including one or more introns) too large to be amplified with the short thermocycling profile (Evans *et al.,* 2013, de Miranda *et al.,* 2013).

**RNA stability assessment**

We used several methods to assess any degradation in the RNA samples during 7 years of storage at -80 ^o^C, between their extraction and purification in Autumn 2016 and their re-use in the current experiment during Autumn 2023. First we submitted a selection of 15 samples with a range of life-stage, infection time-course and RNA concentration/purity profiles to both a fluorometric degradation analysis using the Qubit-4 system (InVitrogen, Thermo Fisher Scientific, Waltham, Massachusetts, USA), and an RNA ScreenTape® microelectrophoresis analysis using the 4150 TapeStation instrument and corresponding analysis software v3.2 (Agilent, Santa Clara, California, USA), as well as determining their concentration and purity with respect to protein (260nm/280nm ratio) and phenolic (260nm/230nm ratio) contamination, and comparing the current results to those obtained in 2016 (Thaduri *et al.,* 2019). Finally, we replicated the original qPCR assays for ABPV, DWV and RP49 mRNA conducted in 2016 (Thaduri *et al.,* 2019) on the newly produced cDNA and ran a CV analysis on the ratio of the new Cq value to the old Cq value. Ratios above 1.00 indicate lower-than-expected performance, which could be due to RNA degradation. Ratios below 1.00 indicate better-than-expected performance, which could be due to higher quality reagents for the new round of RT-qPCR. In this experiment, the average ratio across all 78 samples was slightly above 1.00 for all three assays (1.03 for ABPV, 1.05 for DWV, 1.11 for RP49), with highly uniform figures across all samples for ABPV (CV = 0.053) and RP49 (CV = 0.073) and more variable ratios for DWV (CV = 0.651). These analyses confirmed that, across the experiment, the integrity of these RNA samples was largely conserved over time with only minimal degradation (Table S2, Figure S1), and that the RNA samples retained sufficient quality for a trustworthy gene expression analysis using RT-qPCR.

***De novo* cDNA synthesis and qPCR assays**

For each sample included in the study, the RNA was first diluted to 10 ng/µL with sterile water, following which cDNA was synthesized from this diluted RNA in 200 µL qPCR strip-tubes using the High Capacity cDNA Reverse Transcription Kit and its recommended protocols (ThermoFisher Scientific, Waltham, Massachusetts, USA), with the 20 µL reaction volumes containing 2 µl (20 ng) RNA, 1 µL random hexamer primers, 8 µL water, 4 µL 5XReaction Buffer, 1 µL RiboLock RNase Inhibitor (20 U/µL), 2 µL 10 mM dNTP Mix and 2 µL M-MuLV Reverse Transcriptase (20 U/µL). The strip-tubes were incubated in the Bio-Rad CFX-Connect thermocycler (Bio-Rad, Hercules, California, USA), using a one-step thermocycling profile of 60 min at 37 °C. A working 1/100 dilution cDNA template plate for the qPCR assays was prepared by mixing 2 µL cDNA with 198 µL sterile water. The primary and diluted cDNA samples were stored at -20 °C until further use.

The cDNA of the genes and viruses targeted by the different qPCR assays (Table S1) was amplified and quantified using the Bio-Rad CFX-Connect thermocycler and SsoFast EvaGreen qPCR reaction reagents and detection chemistry (Bio-Rad, Hercules, California, USA). The assays were conducted in 10 µL volumes containing 5 µL of 2× SsoFast EvaGreen Master Mix, 2 µL of diluted cDNA template, 0.3 µL of forward and reverse gene-specific primers (Table S1, 0.5 µM final concentration each), and 2.7 µL nuclease-free water. Since there were 78 samples in the experiment, the qPCR reactions were conducted in a 96-well qPCR assay plate with the experimental samples arranged between columns 2 and 11, leaving columns 1 and 12 for duplicate external dilution standards series specific to each qPCR assay plus non-template negative controls. Two duplicate plates were run for each assay. The thermocycling profile for all assays was as follows: an initial denaturation at 95 °C for 30 seconds, followed by 40 cycles of denaturation at 95 °C for 5 seconds and annealing/extension at 58 °C for 5 seconds. A melt curve analysis was performed immediately after amplification to confirm the identity of the PCR products. The melt curve profile included a 60-second incubation at 95 °C, followed by 60 seconds at 65 °C, and a fluorescence reading taken in 0.5°C increments from 65 °C to 95 °C. For absolute quantification, two 10-fold serial dilution series of a synthetic positive control of known concentration specific to the particular assay were included on each reaction plate, alongside non-template negative controls (water), to generate the external calibration curves, using Bio-Rad CFX software. All positive amplifications were verified for correct product identity by visual inspection of their characteristic melting curves before inclusion in the quantitative analysis. Two replicates were performed on each sample with the average Cq value retained for gene expression analysis. The consistency of the qPCR reactions between the two replicates was determined by calculating the ratio of the duplicate Cq values for each sample. The average of these ratios across samples varied between 0.999 and 1.001 for the different assays testifying to the high level of technical consistency between the duplicate runs of each assay (Supplementary Table S1). Finally, the amplification efficiency (*E*) of each assay was determined from the slope of the regression of Cq value onto the Log_10_ of the concentration of a 7-step 10-fold dilution series of a positive control for the assay (Pfaffl, 2001; Pfaffl et al., 2004), with each dilution series run in quadruplicate, using the same reagents, reaction volumes, thermocycler and cycling profile as for the experimental samples:

Cq*_(g,d)_* = *a_g_* + *b_g_*Log_10_[*dilution_(g,d)_*]	(Eq. 1)

and

*E_g_* = 10^[-1/^*^b^_g_*^]^	(Eq. 2)

Where:

Cq*_(g,d)_*	= Cq value for gene *‘g’* at dilution *‘d’*

*a_g_*	= Y-axis intercept of the linear regression equation for gene *‘g’*

*b_g_*	= Slope of the linear regression equation for gene *‘g’*

*dilution_(g,d)_*	= Concentration *‘d’* of a 10-fold dilution series of gene *‘g’*

*E_g_*	= PCR assay reaction efficiency for gene *‘g’*

**Gene Expression Analysis**

The variation in the levels of gene expression for the various immune genes targeted was analysed using a Relative Quantification approach. In Relative Quantification, the level of a target gene in an experimental sample (ABPV-infected in this study) is compared to the level in a control sample (uninfected, in this study) to determine the effect of the experimental treatment (ABPV infection) on gene expression. Essential for this approach is that the amount of mRNA in the two samples is identical, in order to conclude that the difference is due to a change in gene expression, rather than the amount of mRNA. The ideal tool to represent the entire pool of mRNA, and to normalize the target gene levels for differences between samples in mRNA quantity and quality, is one or more Internal Reference Genes (IRGs), either added exogenously early in the RNA extraction procedures (Forsgren et al., 2017) or as universal, integral and relatively invariant parts of the mRNA pool, whose levels are determined by the same technology as the target genes (RT-qPCR), thus avoiding technology-specific variability (Bustin *et al.,* 2009). The only relevant criterium for a suitable IRG is that its expression levels should be (relatively) invariant across the entire experiment. Common IRGs are therefore those genes involved in universal basal cellular processes, such as energy metabolism, cell structure and protein synthesis. However, depending on the experiment, also these ‘housekeeping’ genes can be subject to expression control and variation in levels (Forsgren et al., 2017). This means that the suitability of all potential IRGs for Normalization purposes needs to be assessed independently for each type of experiment (Bustin *et al.,* 2009, Bustin 2024). There are a number of statistical approaches and tools for determining which gene(s), and how many, are required for reliable Normalization of the target gene data (Andersen et al., 2004; Pfaffl et al., 2004; Vandesompele et al., 2002) producing a distinct subgenre of scientific literature dealing specifically with identifying suitable IRGs for different types of experiments in different host organisms. The one most pertinent for this study is by Deng *et al.,* (2020), who evaluated 11 candidate genes for suitability as IRG for experiments involving IAPV infection in adult honeybees. However, the variability across our experiment in Cq value for our six candidate internal reference genes (RP49, GAPDH, Amrps18, Amtbp, Amtif and Amache2) as recommended by various studies was higher than for many of the recommended immune gene targets (Chen *et al.,* 2014; De Smet *et al.,* 2017, Deng *et al.,* 2020). In this experiment, the least variable target across all samples was the *Cbl* gene, ordinarily part of the JAK/STAT pathway, as determined by a Coefficient of Variation (CV) analysis (Table S1). Accordingly, in this particular experiment Cbl was used as the IRG for Normalizing the sample-specific differences in RNA quantity and quality (Pfaffl, 2001; Pfaffl et al., 2004).

*N_(g,x)_* = *E_i_*^[Cq^*^(i,x)]^* / *E_g_*^[Cq^*^(g,x)]^*^)^	(Eq. 3)

Where:

*N_(g,x)_*	= Normalized amount of immune gene *‘g’* in sample *‘x’*

*E_i_*	= Efficiency of the Internal Reference Gene *‘i’* qPCR assay

Cq*_(i,x)_*	= Cq value of the Internal Reference Gene *‘i’* in sample *‘x’*

*E_g_*	= Efficiency of the immune gene *‘g’* qPCR assay

Cq*_(g,x)_*	= Cq value of immune gene *‘g’* for sample *‘x’*

The Relative Quantification (RQ) of the mRNA levels of the target gene in response to ABPV infection was subsequently calculated as the ratio of the Normalized mRNA levels in the ABPV-inoculated sample to those of the corresponding mock-inoculated sample (Pfaffl, 2001; Pfaffl et al., 2004):

RQ*_g_^(c,t)^* = *N_g_*[ABPV*^(c,t)^*] / *N_g_*[control*^(c,t)^*]	(Eq. 4)

Where:

RQ*_g_^(c,t)^*	= Relative Quantification of immune gene *‘g’* between

ABPV-fed and sugar-fed bees from colony *‘c’* at time-point *‘t’*

*N_g_*[ABPV*^(c,t)^*]	= Normalized amount *‘N’* of immune gene *‘g’* in ABPV-fed

bees from colony *‘c’* at time-point *‘t’*

*N_g_*[control*^(c,t)^*]	= Normalized amount *‘N’* of immune gene *‘g’* in sugar-

fed bees from colony *‘c’* at time-point *‘t’*

We then analysed these RQ values for the different immune genes between the two bee populations and across time population, using the experimental replicates and the different colonies in each population as a source of error variation, to test the significance of the differences in RQ values between populations and across time.

**Additional References**

Andersen, C. L., Jensen, J. L., & Ørntoft, T. F. (2004). Normalization of Real-Time Quantitative Reverse Transcription-PCR Data: A Model-Based Variance Estimation Approach to Identify Genes Suited for Normalization, Applied to Bladder and Colon Cancer Data Sets. *Cancer Research*, *64*(15), 5245–5250. https://doi.org/10.1158/0008-5472.CAN-04-0496

De Miranda, J. R., Bailey, L., Ball, B. V., Blanchard, P., Budge, G. E., Chejanovsky, N., Chen, Y.-P., Gauthier, L., Genersch, E., De Graaf, D. C., Ribière, M., Ryabov, E., De Smet, L., & Van Der Steen, J. J. M. (2013). Standard methods for virus research in *Apis mellifera*. *Journal of Apicultural Research*, *52*(4), 1–56. https://doi.org/10.3896/IBRA.1.52.4.22

Forsgren, E., Locke, B., Semberg, E., Laugen, A. T., & Miranda, J. R. de. (2017). Sample preservation, transport and processing strategies for honeybee RNA extraction: Influence on RNA yield, quality, target quantification and data normalization. *Journal of Virological Methods*, *246*, 81–89. https://doi.org/10.1016/j.jviromet.2017.04.010

Vandesompele, J., De Preter, K., Pattyn, F., Poppe, B., Roy, N., De Paepe, A., & Speleman, F. (2002). Vandesompele J, De Preter K, Pattyn F, Poppe B, Van Roy N, De Paepe A, Speleman F. Accurate normalization of real-time quantitative RT-PCR data by geometric averaging of multiple internal control genes. Genome Biology 3; RESEARCH0034. *Genome Biology*, *3*, RESEARCH0034. https://doi.org/10.1186/gb-2002-3-7-research0034
